# Wünderlich syndrome mimicking acute cholecystitis: a case report

**DOI:** 10.1093/jscr/rjag839

**Published:** 2026-09-20

**Authors:** Juan A Díaz-Esquivel, Flavio J Sánchez-Andrade, Jesús A Payan-Rosete, Christian R Sancho-Hernández, Alberto Sánchez-Lara

**Affiliations:** Department of General Surgery, Hospital General de Tlaxcala “Lic. Anselmo Cervantes Hernández,” Secretaría de Salud, Tlaxcala, Z.C. 90606, México; Department of General Surgery, Hospital General de Tlaxcala “Lic. Anselmo Cervantes Hernández,” Secretaría de Salud, Tlaxcala, Z.C. 90606, México; Department of General Surgery, Hospital General de Tlaxcala “Lic. Anselmo Cervantes Hernández,” Secretaría de Salud, Tlaxcala, Z.C. 90606, México; Department of General Surgery, Hospital General de Tlaxcala “Lic. Anselmo Cervantes Hernández,” Secretaría de Salud, Tlaxcala, Z.C. 90606, México; Department of Anatomic Pathology, Hospital General de Tlaxcala “Lic. Anselmo Cervantes Hernández,” Secretaría de Salud, Tlaxcala, Z.C. 90606, México

**Keywords:** abdominal pain, cholecystitis, hematoma, kidney diseases, tomography

## Abstract

Wünderlich syndrome, or spontaneous non-traumatic renal hemorrhage, is a rare and potentially life-threatening condition. We report the case of a 52-year-old woman presenting with right upper quadrant pain radiating to the lumbar region, bilious vomiting, and positive Murphy’s and Giordano’s signs, initially suggesting acute biliary disease. Ultrasonography demonstrated cholelithiasis and suspected choledocholithiasis; however, computed tomography urography revealed a large right-sided subcapsular and perirenal hematoma associated with a renal pelvic calculus. Because of the hematoma volume and suspicion of malignancy, nephrectomy was performed. Histopathological examination demonstrated an organized subcapsular hematoma with chronic inflammatory changes and no evidence of neoplasia. This case highlights the diagnostic challenge posed by atypical presentations of Wünderlich syndrome and underscores the essential role of computed tomography in distinguishing spontaneous renal hemorrhage from more common causes of acute abdominal pain.

## Introduction

Spontaneous subcapsular or perirenal hemorrhage (Wünderlich syndrome) is an uncommon but potentially life-threatening condition characterized by non-traumatic bleeding into the renal compartment. More than half of reported cases are secondary to renal neoplasms, predominantly renal cell carcinoma and angiomyolipoma [1, 2]. Although the classical presentation consists of Lenk’s triad—acute flank pain, palpable flank mass, and hypovolemic shock—this occurs in only ~20% of patients, making diagnosis challenging [1, 2]. Consequently, computed tomography (CT) is considered the imaging modality of choice because it accurately confirms the diagnosis, determines the extent of hemorrhage, and frequently identifies the underlying etiology [2, 3]. We report an unusual presentation of spontaneous renal hemorrhage initially mimicking acute calculous cholecystitis.

## Case report

A 52-year-old woman with poorly controlled type 2 diabetes mellitus secondary to treatment non-adherence and a history of umbilical hernia repair presented with a 24-hour history of right upper quadrant pain after ingestion of cholecystokinetic foods. The pain radiated to the right lumbar region and was associated with bilious vomiting.

**Figure 1:**
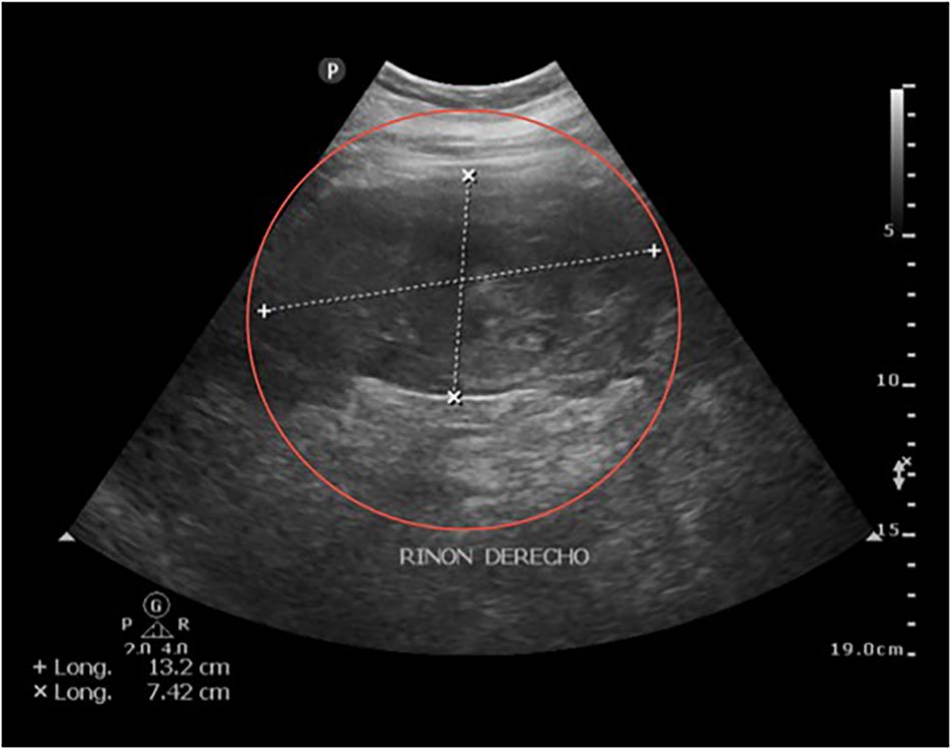
Ultrasonography. Right kidney compressed by a heterogeneous mass (circle). *Source: Image obtained by the authors.*

**Figure 2:**
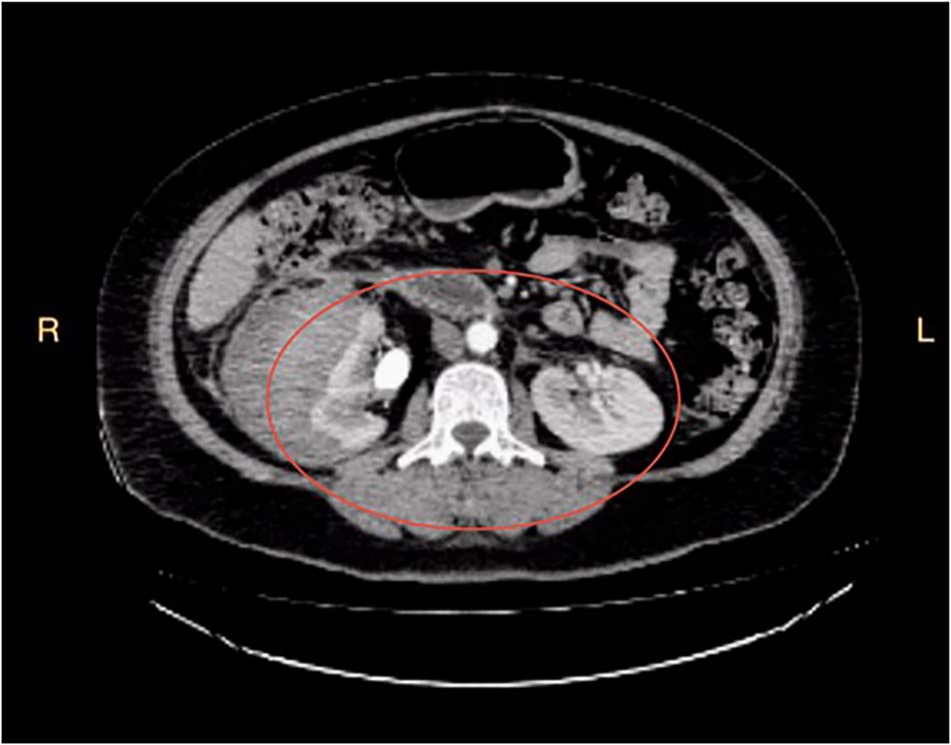
CT urography (axial view). Right kidney demonstrating a homogeneous parenchyma, adjacent to an irregular, heterogeneous mass with predominant hyperdensity compressing the renal parenchyma. A well-defined oval, hyperdense lesion is visible within the renal pelvis (circle). *Source: Image obtained by the authors.* Adjacent to this lesion, a well-defined, irregular, heterogeneous retroperitoneal mass was identified (45 HU) with alternating zones of fluid density (11 HU) and an estimated volume of 577 cc, exerting a significant mass effect on the right kidney and associated with mild perirenal fat stranding.

Physical examination revealed stable vital signs, right upper quadrant tenderness with a positive Murphy sign, right costovertebral angle tenderness, and fullness of the right renal fossa without signs of peritoneal irritation. Laboratory evaluation demonstrated hemoglobin of 10.9 g/dL, leukocytosis (15.48 × 10^3^/μL), and severe hyperglycemia (468 mg/dL). Abdominal ultrasonography demonstrated multiple gallstones and a hyperechoic image initially interpreted as possible choledocholithiasis. Evaluation of the right kidney revealed heterogeneous renal parenchyma with loss of corticomedullary differentiation, prompting further investigation. CT urography identified a right renal pelvic calculus and a large heterogeneous right perirenal retroperitoneal collection exerting marked mass effect on the kidney with associated mild perirenal fat stranding, consistent with a large hemorrhagic collection (Figs 1 and 2).

Given the diagnosis of chronic calculous cholecystitis requiring surgical treatment and the presence of a massive perirenal hematoma with a high suspicion of underlying malignancy, the patient underwent glycemic optimization with continuous intravenous insulin infusion followed by open cholecystectomy. A Kocher maneuver and partial Cattell-Braasch maneuver were subsequently performed to expose the retroperitoneum. Prominent paracaval lymphadenopathy prompted regional lymphadenectomy, followed by right radical nephrectomy because of the extensive hematoma, the risk of recurrent hemorrhage, the associated staghorn calculus with high predisposition to infection, and the inability to exclude renal malignancy preoperatively. An open approach was selected because laparoscopic equipment was unavailable and the size of the specimen precluded minimally invasive extraction.

**Figure 3:**
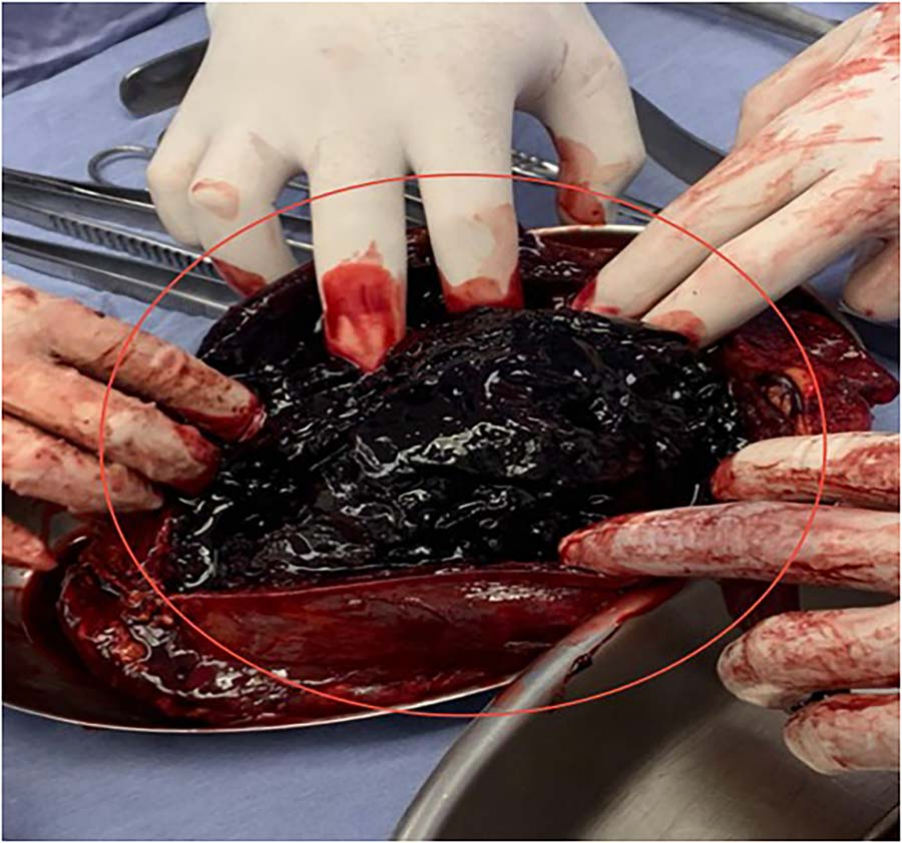
Gross pathology. Semi-solid mass with an estimated volume of 600 cc (circle). *Source: Photograph taken by the authors.*

Gross pathology examination revealed a right kidney measuring 25 × 30 × 18 cm with a large violaceous mass arising from the parenchyma, a 3-cm renal pelvic staghorn calculus, and paracaval lymph nodes showing marked inflammatory features. The excised gallbladder contained multiple calculi smaller than 1 cm. Sectioning of the kidney demonstrated extensive coagulated blood and severe disruption of the parenchymal architecture (Fig. 3).

**Figure 4:**
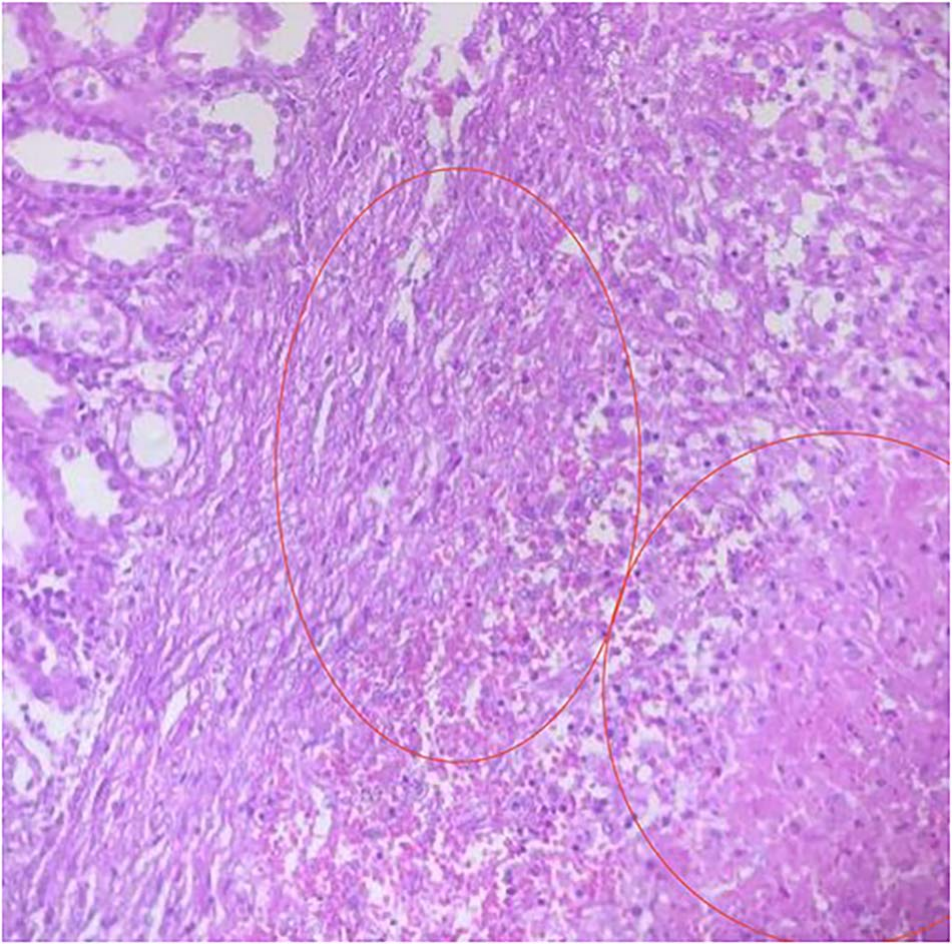
Histopathological evaluation. The boundaries of the subcapsular hematoma are identified at the renal periphery. The right side of the section demonstrates fragmented erythrocytes, cellular debris, neutrophilic polymorphonuclear infiltrates, and fibrin deposition. Centrally, fibrohistiocytic proliferation secondary to hematoma organization is observed, indicating chronicity (circle). *Stain: Hematoxylin–Eosin (H&E). Scale: 20×. Source: Photograph taken by the authors.*

Histopathological evaluation reported a subcapsular and cortical renal hematoma, acute-on-chronic ischemic glomerulonephritis, acute tubular necrosis, and focal segmental glomerulosclerosis. Microscopic analysis (Fig. 4) clearly delineated the boundaries of the subcapsular hematoma at the renal periphery with fragmented erythrocytes, cellular debris, fibrin deposition, and a dense neutrophilic polymorphonuclear infiltrate. Centrally, prominent fibrohistiocytic proliferation secondary to hematoma organization was identified, confirming the chronic nature of the hemorrhagic event.

The postoperative course was uneventful, and the patient was discharged on postoperative Day 6. At 3-month follow-up, she remained asymptomatic with preserved renal function.

## Discussion

Spontaneous renal hemorrhage, or Wünderlich syndrome, is an uncommon clinical entity with a broad spectrum of presentations, frequently delaying diagnosis. Although acute flank pain is the predominant symptom, the complete Lenk’s triad is present in only a minority of patients, limiting its clinical utility [2, 4]. Consequently, the differential diagnosis should be broadened when abdominal pain is accompanied by unexplained anemia or discordant imaging findings.

The distinguishing feature of the present case was the ability of a large right-sided perirenal hematoma to clinically and radiologically mimic acute calculous cholecystitis. Right upper quadrant pain, bilious vomiting, a positive Murphy sign, and concomitant cholelithiasis strongly supported an initial biliary diagnosis. Moreover, ultrasonography suggested choledocholithiasis, illustrating the limitations of this modality when large retroperitoneal collections distort normal anatomical relationships. CT urography accurately characterized the hemorrhage, excluded biliary obstruction, and provided the anatomical information required for definitive surgical management [2, 3, 5].

Renal neoplasms account for most reported cases of spontaneous renal hemorrhage, with angiomyolipoma and renal cell carcinoma representing the most frequent benign and malignant etiologies, respectively [1, 2, 4, 6]. Nevertheless, vascular abnormalities, infectious processes, chronic kidney disease, anticoagulant therapy, hereditary cystic disorders, and obstructive uropathy should also be considered [6–9]. In our patient, the large hemorrhagic renal lesion and associated paracaval lymphadenopathy raised a strong preoperative suspicion of malignancy; however, histopathological examination demonstrated an organized subcapsular hematoma associated with severe chronic renal injury without evidence of neoplasia.

An additional clinicopathological observation was the discrepancy between the abrupt onset of symptoms and the chronic histological features of the hematoma. Fibrohistiocytic proliferation, fibrin deposition, and hematoma organization indicated that the hemorrhagic process preceded the acute presentation, suggesting progressive enlargement until sufficient mass effect and local inflammatory irritation developed to reproduce the clinical features of acute biliary disease.

## Conclusions

Wünderlich syndrome should be considered in the differential diagnosis of acute abdominal pain, even in the absence of Lenk’s triad, particularly when clinical and ultrasonographic findings are incongruent. CT remains the cornerstone for diagnosis and surgical planning, whereas histopathological evaluation is essential to establish the underlying etiology. This case expands the spectrum of atypical presentations of spontaneous renal hemorrhage by demonstrating its ability to mimic acute calculous cholecystitis.
